# Neuromedin U and Its Putative Drosophila Homolog hugin

**DOI:** 10.1371/journal.pbio.0040068

**Published:** 2006-03-14

**Authors:** Christoph Melcher, Rüdiger Bader, Steffen Walther, Oleg Simakov, Michael J Pankratz

**Affiliations:** **1**Forschungszentrum KarlsruheKarlsruheGermany

We recently provided a molecular, neuroanatomical, and behavioral analyses of neurons expressing the neuropeptide gene hugin in Drosophila [1]. hugin-expressing neurons appear to comprise a neural circuitry in the brain that modulates feeding behavior in response to gustatory and nutrient signals. One of the questions arising from this study was whether a mammalian homolog of hugin exists. We argue here that a mammalian homolog of Drosophila hugin may be neuromedin U (NmU).

NmU was originally isolated from porcine spinal cord based on its ability to contract uterine smooth muscle [2]. Characterization of porcine NmU identified two peptides with similar bioactivity, a 25-mer (NmU-25) and an 8-mer (NmU-8). NmU-8 is derived from cleavage of NmU-25 and shares an identical C-terminus, which is critical for bioactivity, and is highly conserved among vertebrates [2].

One of the peptides produced by the Drosophila hugin gene is pyrokinin-2 (PK-2), which also possesses myostimulatory activity [3]. This peptide also bears striking sequence resemblance to mammalian NmU-8. Both are 8-mers, and porcine NmU-8 sequence (YFLFRPRN) and Drosophila PK-2 sequence (SVPFKPRL) share three of eight amino acid residues. The three common residues lie in the last five amino acids; among the vertebrates, the last five residues are identical. Cockroach, Periplaneta americana, pyrokinin sequence (LVPFRPRL) [4] shows even higher homology to porcine NmU-8, with four of eight amino acids being identical, again all in the last five residues. Putative G-protein-coupled receptors for Drosophila PK-2 also share high homology with mammalian NmU receptors [5,6].

The structure of the prepropeptides that gives rise to mammalian NmU-8 and Drosophila PK-2 is also similar. Human and rat NmU genes, and hugin, encode prepropeptides that can be potentially cleaved into three peptides [3,7]. Nmu-8 and Drosophila PK-2 are derived from the last peptide. In Drosophila, the middle peptide was termed hugin-&gamma; [3]. Whether other cleavage products from vertebrates encode functional neuropeptides remains to be determined, but the high conservation between rat and human sequences in this region (36 of 38 identical amino acids) suggests an important function [7].

Similarities between NmU and hugin extend to the functional level. Rat NmU is specifically expressed in the ventromedial hypothalamus, a region involved in regulating feeding, and its expression is downregulated upon fasting [8]; hugin is specifically expressed in the subesophageal ganglion, a brain region in Drosophila regulating feeding, and its expression is also downregulated upon starvation [1]. Administration of NmU causes suppression of feeding in rats [8], while NmU knockout in mice causes hyperphagia [9]; in Drosophila, overexpression of hugin causes suppression of growth and feeding [1,3], while blocking synaptic activity of hugin neurons causes increased feeding [1].

Based on these considerations, we propose that Drosophila hugin may be a homolog of mammalian NmU. Future experiments and data comparisons should provide more insights into this issue.
